# Imaging and targeted therapy of pancreatic ductal adenocarcinoma using the theranostic sodium iodide symporter (NIS) gene

**DOI:** 10.18632/oncotarget.16499

**Published:** 2017-03-23

**Authors:** Kathrin A. Schmohl, Aayush Gupta, Geoffrey K. Grünwald, Marija Trajkovic-Arsic, Kathrin Klutz, Rickmer Braren, Markus Schwaiger, Peter J. Nelson, Manfred Ogris, Ernst Wagner, Jens T. Siveke, Christine Spitzweg

**Affiliations:** ^1^ Department of Internal Medicine II and IV, University Hospital of Munich, LMU Munich, Munich, Germany; ^2^ Department of Internal Medicine II, Klinikum rechts der Isar der Technischen Universität München, Munich, Germany; ^3^ Division of Solid Tumor Translational Oncology, West German Cancer Center, University Hospital Essen, Essen, Germany; ^4^ German Cancer Consortium (DKTK), Partner Site Essen and German Cancer Research Center (DKFZ), Heidelberg, Germany; ^5^ Department of Radiology, Klinikum rechts der Isar der Technischen Universität München, Munich, Germany; ^6^ Department of Nuclear Medicine, Klinikum rechts der Isar der Technischen Universität München, Munich, Germany; ^7^ Clinical Biochemistry Group, Department of Internal Medicine IV, University Hospital of Munich, LMU Munich, Munich, Germany; ^8^ Department of Pharmaceutical Chemistry, Laboratory of MacroMolecular Cancer Therapeutics (MMCT), University of Vienna, Vienna, Austria; ^9^ Pharmaceutical Biotechnology, Department of Pharmacy, Center for System-Based Drug Research and Center for Nanoscience, LMU Munich, Munich, Germany

**Keywords:** gene therapy, sodium iodide symporter, EGFR-targeting, pancreatic ductal adenocarcinoma, genetically engineered mouse model

## Abstract

The theranostic sodium iodide symporter (NIS) gene allows detailed molecular imaging of transgene expression and application of therapeutic radionuclides. As a crucial step towards clinical application, we investigated tumor specificity and transfection efficiency of epidermal growth factor receptor (EGFR)-targeted polyplexes as systemic *NIS* gene delivery vehicles in an advanced genetically engineered mouse model of pancreatic ductal adenocarcinoma (PDAC) that closely reflects human disease. PDAC was induced in mice by pancreas-specific activation of constitutively active *Kras^G12D^* and deletion of *Trp53*. We used tumor-targeted polyplexes (LPEI-PEG-GE11/NIS) based on linear polyethylenimine, shielded by polyethylene glycol and coupled with the EGFR-specific peptide ligand GE11, to target a *NIS*-expressing plasmid to high EGFR-expressing PDAC. *In vitro* iodide uptake studies in cell explants from murine EGFR-positive and EGFR-ablated PDAC lesions demonstrated high transfection efficiency and EGFR-specificity of LPEI-PEG-GE11/NIS. *In vivo* ^123^I gamma camera imaging and three-dimensional high-resolution ^124^I PET showed significant tumor-specific accumulation of radioiodide after systemic LPEI-PEG-GE11/NIS injection. Administration of ^131^I in LPEI-PEG-GE11/NIS-treated mice resulted in significantly reduced tumor growth compared to controls as determined by magnetic resonance imaging, though survival was not significantly prolonged. This study opens the exciting prospect of NIS-mediated radionuclide imaging and therapy of PDAC after systemic non-viral *NIS* gene delivery.

## INTRODUCTION

Pancreatic ductal adenocarcinoma (PDAC) is currently the fourth leading cause of cancer-related mortality in developed countries despite its comparably low incidence of less than 3 %, clearly demonstrating the lack of effective therapeutic strategies. The five-year survival rate is around 7 % for all stages of the disease and drops to below 2 % and a median survival of less than a year for patients with metastatic disease, mostly due to late diagnosis at the stage of inoperability and the unusually high resistance of PDAC to conventional radiation and chemotherapy [1, 2]. Despite intensive scientific and industrial efforts, so far no significant extension of survival could be achieved by any of the numerous therapy approaches tested [3].

The genetic and morphological changes in the carcinogenesis of PDAC are well known and include the initiation and progression of premalignant lesions to invasive and metastatic pancreatic cancer [3–6]. The genetic hallmark of PDAC development is an activating mutation in the *KRAS* oncogene, followed by other genetic changes, commonly including inactivation of the tumor suppressors *TP53*, *CDKN2A* (*P16^INK4A^*) and *SMAD4*, and activation of several growth factor receptors, such as the epidermal growth factor receptor (EGFR) [3, 7].

Against this background, several complex genetically modified mouse models of PDAC that mirror the typical changes found in human patients, have been generated in recent years [3, 4, 8–10]. One such model is the *Ptf1a^+/Cre^;Kras^+/LSL-G12D^;Trp53^loxP/loxP^ (Kras;p53)* mouse. Here, PDAC is induced by pancreas-specific activation of constitutively active *Kras^G12D^* in combination with conditional deletion of *Trp53* [8]. To restrict these genetic modifications to the pancreas, mice with the mutated alleles are interbred with animals that express the Cre recombinase driven by the pancreas-specific promoter for Ptf1a, a subunit of pancreas transcription factor 1 (Ptf1) that is required to commit cells to a pancreatic fate during embryonic development [4, 11]. Thus, the activation of the oncogenic *Kras^G12D^* via excision of a transcriptionally inhibitory *LSL* (*loxP-STOP-loxP*) construct and deletion of the floxed tumor suppressor *Trp53* occur in the pancreas only, leading to ductal lesions with complete penetrance [4, 12]. The development of endogenous mouse models away from the usual transplant models represents a significant step in the evolution of preclinical models [13]. The morphological and molecular composition of endogenous tumors far better reflects human disease, making them highly suitable to predict the clinical effectiveness of a specific treatment strategy.

The sodium iodide symporter (NIS; *SLC5A5*) mediates the uptake of iodide into thyroid follicular cells allowing both diagnostic and therapeutic application of radioiodide in thyroid cancer patients [14, 15]. In our previous work, we have extensively investigated the dual reporter/therapy capacity of NIS in various non-thyroidal tumors and have proven the feasibility of extrathyroidal radioiodide therapy after tumor-selective *NIS* gene transfer [16–26]. Transfection of cancer cells with the *NIS* gene allows non-invasive monitoring of functional NIS expression and *in vivo* biodistribution before the application of a therapeutic dose of radioiodide. One of the major hurdles of efficient and safe application of the *NIS* gene therapy concept in the clinical setting is optimal tumor-specific targeting in the presence of low toxicity and high transfection efficiency of gene delivery vectors, with the ultimate goal of systemic vector application.

In a previous study, we used synthetic polyplexes based on pseudodendritic oligoamines with high intrinsic tumor affinity for *NIS* gene therapy in a syngeneic neuroblastoma mouse model as well as a subcutaneous human hepatocellular carcinoma mouse model [16, 18]. After systemic *NIS* gene transfer, the tumor-selective accumulation of radioiodide was sufficient for a significant therapeutic effect. In addition to an intrinsic tumor affinity due to the so-called enhanced permeability and retention (EPR) effect based on “leaky” tumor vasculature, the tumor targeting of polyplexes can be further optimized by the attachment of tumor-specific ligands. To this end, in a subsequent study, we used LPEI-PEG-GE11 polymers composed of linear polyethylenimine (LPEI), shielded by polyethylene glycol (PEG) and coupled to the synthetic peptide GE11 as an EGFR-specific ligand for *NIS* gene delivery [17]. After systemic application of these polymers condensed with *NIS* DNA, tumor-specific radioiodide accumulation demonstrated effective and EGFR-specific tumor targeting in a high EGFR-expressing xenograft mouse model of hepatocellular carcinoma. After the injection of a therapeutic dose of ^131^I, tumoral iodide uptake was sufficiently high for a significant delay of tumor growth and prolongation of animal survival [17].

Based on our previous work and the well-known characteristic upregulation of EGFR in PDAC, we investigated the potential of EGFR-targeted polyplexes for systemic *NIS* gene therapy in an advanced endogenous mouse model of PDAC as a next step towards clinical application.

## RESULTS

### Iodide uptake studies *in vitro*

**Figure 1:**
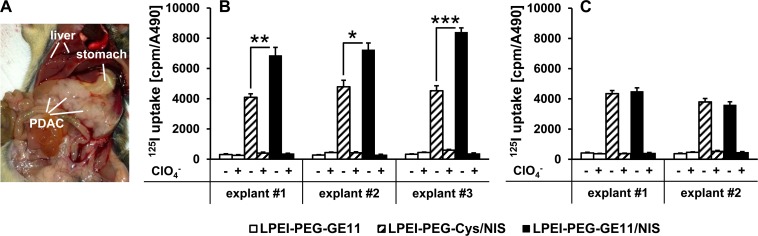
Iodide uptake in PDAC cell explants *in vitro* *Kras;p53* mice develop PDAC that occupies a large portion of the abdominal cavity below the stomach **(A). (B)** PDAC cell explants from three separate mice (three technical replicates per mouse) transfected *in vitro* with LPEI-PEG-GE11/NIS showed a significant increase in perchlorate- (ClO_4_^−^-) sensitive ^125^I accumulation compared to transfection with LPEI-PEG-Cys/NIS (mean ± S.E.M.; *p<0.05; **p<0.01; ***p<0.001). No iodide uptake above background levels was observed in cells transfected with LPEI-PEG-GE11 alone. **(C)** Transfection of EGFR-ablated PDAC cell explants from two mice (three technical replicates per mouse) with LPEI-PEG-GE11/NIS and LPEI-PEG-Cys/NIS showed no significant differences between transfection with targeted or untargeted polyplexes, demonstrating EGFR-specificity of the targeting ligand GE11 (mean ± S.E.M.).

In order to optimize transfection conditions for LPEI-PEG-GE11 polymers condensed with the *NIS* plasmid (LPEI-PEG-GE11/NIS) in high EGFR-expressing PDAC cell explants derived from *Kras;p53* mice (Figure 1A), radioiodide uptake activity was evaluated 24 h after polyplex application (data not shown). A conjugate to plasmid (c/p) ratio of 0.8 resulted in highest transfection efficiency at lowest cytotoxicity. Therefore, this c/p ratio was used in all subsequent experiments. Twenty-four hours after transfection with LPEI-PEG-GE11/NIS, cell explants from three different mice showed a 22-26-fold increase in ^125^I accumulation as compared to cells incubated with the empty control vector LPEI-PEG-GE11 (Figure 1B). Transfection with untargeted LPEI-PEG-Cys/NIS (targeting ligand GE11 replaced by a cysteine residue) resulted in significantly lower iodide uptake activity compared to EGFR-targeted LPEI-PEG-GE11/NIS (Figure 1B). In both cases, iodide uptake was blocked upon additional treatment with the NIS-specific inhibitor perchlorate.

To further verify EGFR-specificity of the targeting ligand GE11, we performed additional iodide uptake studies in EGFR-ablated PDAC cell explants from *Ptf1a^+/Cre^;Kras^+/LSL-G12D^;Trp53^loxP/loxP^;Egfr^fl/fl^* (*Kras;p53;Egfr*) mice. No significant difference between transfection with targeted LPEI-PEG-GE11/NIS or untargeted LPEI-PEG-Cys/NIS polyplexes was observed (Figure 1C). Polyplex-mediated *NIS* gene transfer did not affect cell viability for any of the treatment conditions compared to untreated cells as measured by MTS assay (data not shown).

### ^123^I scintigraphy and ^124^I PET imaging of EGFR-targeted NIS gene delivery

Functional NIS expression in mice with high EGFR-expressing PDAC was imaged by whole body ^123^I gamma camera and ^124^I PET imaging. Polyplexes were administered intravenously (i.v.) either 24 or 48 h before injection of the respective radionuclide for imaging.

**Figure 2:**
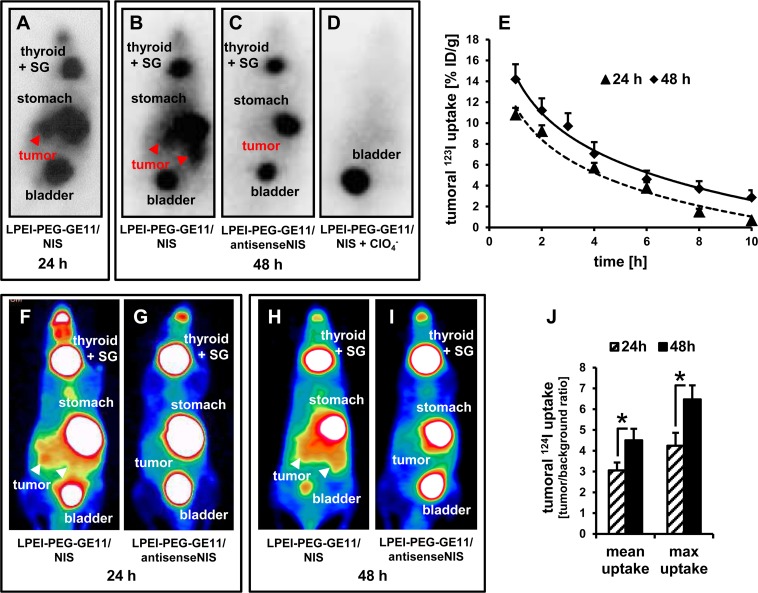
*In vivo* imaging of NIS-mediated iodide uptake ^123^I scintigraphy revealed pancreatic tumoral radioiodide uptake 24 h **(A)** and 48 h **(B)** after injection of mice with LPEI-PEG-GE11/NIS that was not seen after injection with non-coding LPEI-PEG-GE11/antisenseNIS **(C)** Iodide uptake was perchlorate-sensitive **(D)** and therefore indeed NIS-mediated. **(E)** Radionuclide retention time in tumors was determined by serial scanning over 10 h (mean ± S.E.M.; 24 h: n=9, 48 h: n=7). ^124^I PET-imaging confirmed findings of scintigraphy and allowed better differentiation between tumoral and stomach radioiodide uptake **(F, H)** After injection of the control vector LPEI-PEG-GE11/antisenseNIS **(G, I)**, no pancreatic iodide uptake activity above background levels could be detected. Significantly higher radioiodide accumulation 48 h after gene transfer as compared to 24 h was confirmed by PET (mean ± S.E.M.; n=5 each; *p<0.05) **(J)** SG: salivary glands.

*In vivo* ^123^I gamma camera imaging revealed high levels of NIS-mediated radionuclide accumulation in pancreatic tumors both at 24 and 48 h after systemic injection of EGFR-targeted LPEI-PEG-GE11/NIS (Figures 2A, 2B). Tumors accumulated 10.8 ± 0.7 % of the injected dose per gram (ID/g) with an average biological half-life of 4 h at 24 h and 14.2 ± 1.4 % ID/g with an average biological half-life of 4.5 h at 48 h (Figure 2E). For ^131^I, a tumor-absorbed dose of 74.7 mGy/MBq/g tumor with an effective half-life of 3.2 h (24 h after polyplex administration) and 96.5 mGy/MBq/g tumor, effective half-life 4.5 h (48 h after polyplex administration), was calculated. In contrast, injection of non-coding control polyplexes LPEI-PEG-GE11/antisenseNIS (*NIS* sequence back to front) resulted in no significant tumoral radioiodide accumulation (Figure 2C). In addition to ^123^I uptake in the tumor, radioiodide accumulation was also observed in the stomach, the thyroid and the salivary glands, as they physiologically express NIS, as well as in the urinary bladder due to renal radionuclide elimination (Figures 2A-2C). To further confirm that tumoral iodide uptake was indeed NIS-mediated, LPEI-PEG-GE11/NIS-injected mice were additionally treated with the competitive NIS-inhibitor perchlorate 30 min before ^123^I administration, which completely blocked polyplex-mediated tumoral iodide accumulation in addition to physiological uptake in the stomach, the thyroid gland and the salivary glands (Figure 2D).

To better distinguish tumoral uptake from iodide accumulation in the stomach, we additionally employed three-dimensional high-resolution ^124^I PET to image radioiodide biodistribution. Again, systemic injection of LPEI-PEG-GE11/NIS resulted in strong transfection of tumor tissue at both time points (Figures 2F, 2H), an effect that was not seen in mice treated with LPEI-PEG-GE11/antisenseNIS (Figures 2G, 2I). Quantification of tumoral ^124^I uptake again revealed significantly higher radioiodide accumulation 48 h after i.v. injection of LPEI-PEG-GE11/NIS as compared to 24 h after NIS gene transfer (Figure 2J).

### *Ex vivo* analysis of NIS expression in PDAC

**Figure 3:**
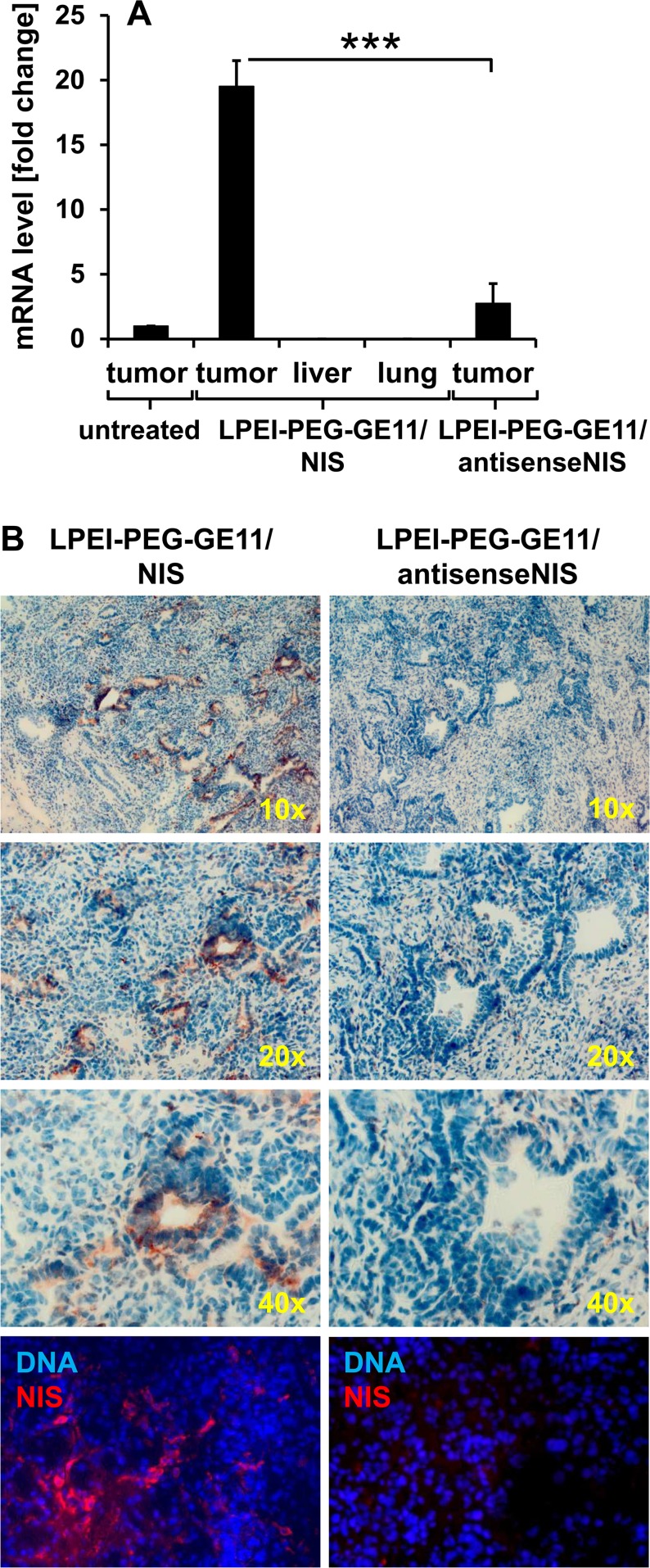
Analysis of NIS mRNA and protein distribution *ex vivo* NIS-specific qPCR analysis revealed a 20-fold increase of *NIS* mRNA expression in pancreatic tumors of mice injected with LPEI-PEG-GE11/NIS as compared to tumors of untreated mice. In contrast, *NIS* mRNA was not increased in non-target organs and in tumors of mice injected with the control vector LPEI-PEG-GE11/antisenseNIS (mean-fold change ± S.E.M.; ***p<0.001) **(A)** Both immunohistochemical (**B**, upper three panels; magnification: 10×, 20× and 40×) and immunofluorescence staining (**B**, bottom panel; magnification: 200×) of sections of pancreatic tumors revealed areas of NIS-specific immunoreactivity after systemic application of LPEI-PEG-GE11/NIS. In contrast, tumors treated with the control vector LPEI-PEG-GE11/antisenseNIS showed no NIS-specific immunoreactivity.

48 h after polyplex administration, mice were sacrificed and dissected. Tumors and non-target organs (liver, lung) were analyzed for *NIS* mRNA expression by quantitative real-time PCR (qPCR). A 20-fold increase in *NIS* mRNA expression was detected in PDAC lesions from mice injected with LPEI-PEG-GE11/NIS as compared to untreated tumors (Figure 3A). In contrast, no significant *NIS* mRNA expression above background levels was observed in non-target organs and tumors of mice treated with the control vector LPEI-PEG-GE11/antisenseNIS (Figure 3A). In tumors from LPEI-PEG-GE11/NIS-treated mice, areas of NIS-specific immunoreactivity were observed surrounding ductal lesions by immunohistochemical and immunofluorescence staining using a human NIS-specific antibody (Figure 3B). NIS staining was found to be both cell membrane-associated and cytoplasmic. In contrast, tumors from mice treated with the control vector LPEI-PEG-GE11/antisenseNIS showed no NIS-specific immunoreactivity (Figure 3B).

### NIS-mediated ^131^I therapy of PDAC

**Figure 4:**
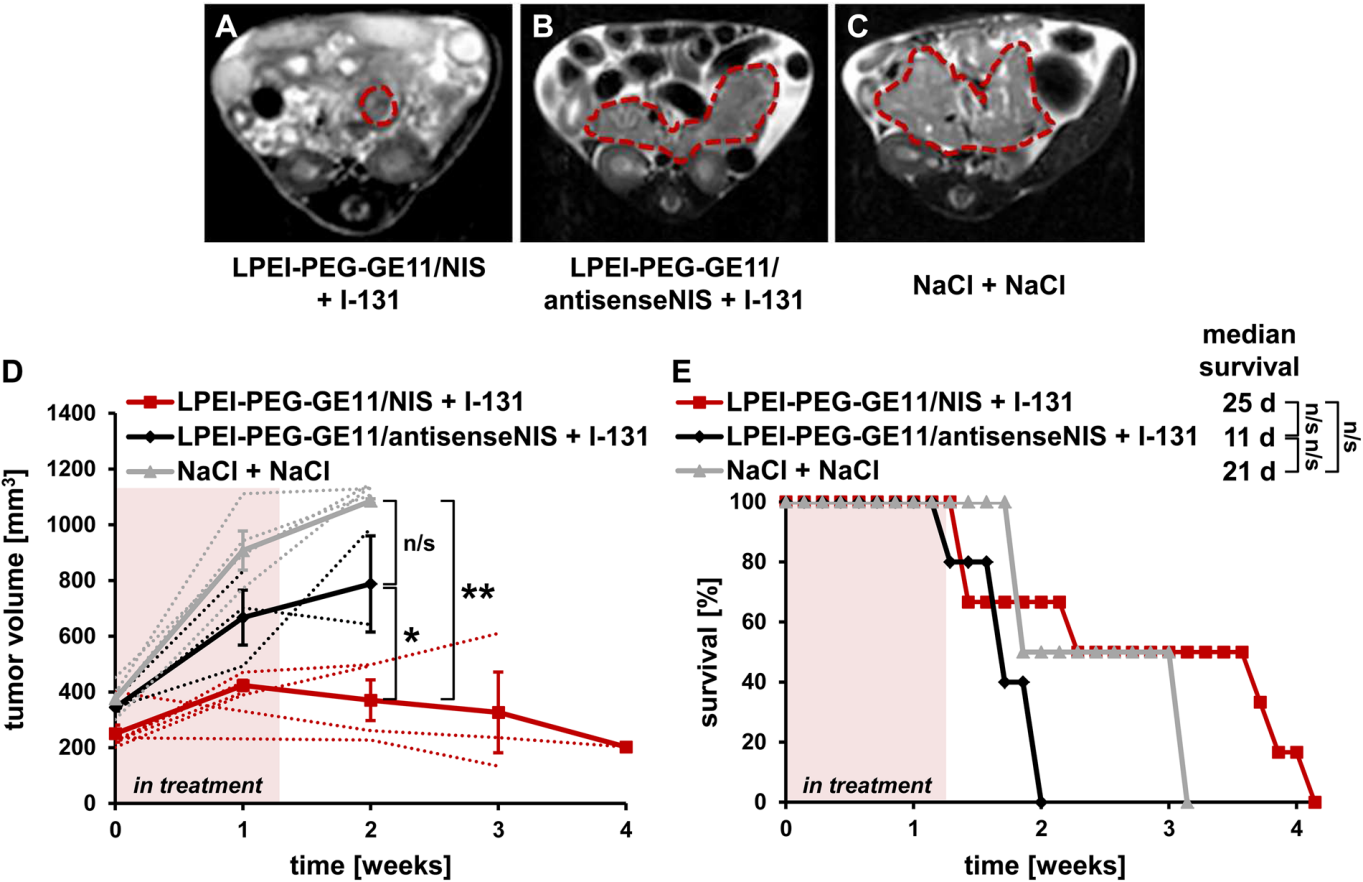
Therapeutic application of 131I after NIS gene transfer *in vivo* *Kras;p53* mice were treated with three cycles of i.v. injection of polyplexes on days 0/4/7 followed by i.p. injection of 55.5 MBq ^131^I 48 h later, on days 2/6/9. Tumor sizes were monitored weekly by MRI. Exemplary MRI images of endpoint tumor sizes from an LPEI-PEG-GE11/NIS + ^131^I- **(A)**, an LPEI-PEG-GE11/ antisenseNIS + ^131^I- **(B)** and a NaCl + NaCl-treated *Kras;p53* mouse are shown **(C)**. Tumors are highlighted by red dotted lines. **(D)** Mice treated with LPEI-PEG-GE11/NIS + ^131^I (n=6) showed a stabilization in tumor volume compared to control groups LPEI-PEG-GE11/antisenseNIS + ^131^I (n=3; mean ± S.E.M.; *p<0.05) and NaCl + NaCl (n=4; **p<0.01). Mean tumor volumes (solid lines) and volumes for individual mice (dotted lines) are shown. **(E)** Injection of LPEI-PEG-GE11/NIS + ^131^I led to an increased overall and median survival in the therapy group (n=6) compared to control groups injected with LPEI-PEG-GE11/antisenseNIS + ^131^I (n=3; n/s) or NaCl + NaCl (n=4; n/s).

PDAC-bearing mice were treated with three cycles of LPEI-PEG-GE11/NIS followed by ^131^I 48 h later – the optimal time point for radionuclide injection based on the imaging studies. Controls were injected with non-coding LPEI-PEG-GE11/antisenseNIS and ^131^I or saline only. Tumor progression was monitored by magnetic resonance imaging (MRI). Mice in the therapy group showed a significant stabilization of tumor growth and, in two cases, even a reduction in tumor volume (Figures 4A, 4D), while aggressive tumor growth was observed in both control groups (Figures 4B-4D). This led to an enhanced survival in the therapy group that lived up to 28 days post therapy start with a median survival of 25 days, as compared to the antisenseNIS group that survived up to 13 days with a median survival of 11 days and saline controls that lived up to 21 days, median survival 21 days (Figure 4E). The effect on mouse survival was, however, not significant.

## DISCUSSION

While the incidence of PDAC is gradually increasing, the prognosis of patients with pancreatic cancer has not significantly changed over the last 20 years – despite numerous advances in diagnostic imaging, surgical techniques and chemotherapeutic strategies [3, 27]. Intensified chemotherapy protocols in patients with advanced pancreatic cancer show a significant, yet still unsatisfactory survival benefit [28]. So far, no targeted agent or approach has changed this fatal course of the disease, even though preclinical trials in *in vitro* cell culture systems and *in vivo* xenograft models had shown promising results [3, 28]. These set-backs can mainly be attributed to the complexity of the disease. The homogeneous molecular equipment, simple stromal architecture and immune deficiency of xenograft models limits their transferability to the clinical setting. Endogenously grown tumors, in contrast, are genetically and morphologically heterogeneous, less vascularized and harbor a far more complex microenvironment with high immunosuppression and extensive desmoplasia [29, 30]. Genetically engineered mouse models that closely reflect the key aspects of pancreatic carcinogenesis have been shown to correlate well with data from clinical trials and provide an exciting new platform to predict human tumor responses to treatment [13].

After the proof-of-principle of our polyplex-mediated *NIS* gene therapy concept in different subcutaneous xenograft tumor models [16–18], the genetically engineered mouse model of PDAC used in this study provides an important step towards further development towards clinical application. Based on the known activity of EGFR in PDAC and this model, we chose EGFR-targeted LPEI-PEG-GE11 polymers as delivery vehicles for the *NIS* gene [17, 31, 32].

Transfection of PDAC explant cell cultures with LPEI-PEG-GE11/NIS led to significant perchlorate-sensitive and therefore NIS-mediated radioiodide accumulation. The empty vector LPEI-PEG-GE11 did not result in iodide accumulation above background levels, further confirming NIS-dependency of radioiodide accumulation. Iodide uptake was significantly reduced after transfection with non-targeted LPEI-PEG-Cys/NIS, demonstrating improved transfection efficiency using the targeting ligand GE11. EGFR-specificity of targeting was further substantiated by the observation that in EGFR-negative cultures derived from *Kras;p53;Egfr* mice, no significant difference between transfection with EGFR-targeted or non-targeted vectors was observed. Translating these promising *in vitro* results to systemic vector application *in vivo*, intravenous administration of LPEI-PEG-GE11/NIS resulted in a significant perchlorate-sensitive tumor-specific iodide uptake in mice harboring endogenous PDAC tumors, as demonstrated by ^123^I gamma camera imaging. Three-dimensional ^124^I PET imaging with increased sensitivity and resolution was employed for more accurate quantification of tumoral radioiodide uptake, as radionuclide signals from pancreatic lesions partially overlap with stomach signals based on physiological gastric NIS expression. Results from PET imaging confirmed gamma camera imaging results with strong radioiodide signals in pancreatic tumors. Control experiments with LPEI-PEG-GE11/antisenseNIS showed no significant tumoral radioiodide accumulation above background levels, confirming NIS-specificity of tumoral tracer uptake. These molecular imaging data were further corroborated by NIS-specific immunohistochemistry and immunofluorescence as well as qPCR analysis.

Both the abundance and the permeability of the tumor's vasculature are crucial for sufficient transgene delivery into the tumor [33, 34]. One of the main factors thought to hamper efficient drug delivery to PDAC, is its highly desmoplastic stroma alongside its high interstitial pressure and poor vascularization [35]. Thus, the enhanced permeability and retention effect that is caused by the irregular, “leaky” tumor vasculature and is usually exploited for passive targeting of therapeutic agents to tumor sites, is limited in PDAC [34, 36]. For this reason, an additional tumor-targeting strategy is particularly important. Our imaging data convincingly demonstrate that targeting our polyplexes to EGFR allows strong transfection of pancreatic tumors with *NIS*. In a previous study, using the same vector construct in a subcutaneous hepatocellular carcinoma xenograft model, a tumor-absorbed dose of 47 mGy/MBq/g was calculated for ^131^I 24 h after polyplex administration [17], while in the current study, a dose of 74.7 mGy/MBq/g tumor 24 h post polyplex injection was achieved. We mainly attribute this significantly enhanced tumoral radioiodide uptake to the very high EGFR expression in PDAC. NIS staining was restricted to areas of high EGFR expression surrounding ductal lesions [7]. This focal pattern of transgene expression further underlines the advantage of NIS as therapy gene in this setting, as the high radionuclide bystander effect allows destruction of tumor cells beyond transfected cells.

Building on these promising results, the next logical step was to evaluate the therapeutic effectiveness of ^131^I in the PDAC mouse model after LPEI-PEG-GE11-mediated systemic *NIS* gene delivery. We were able to demonstrate stabilization, and, in two cases, even a pronounced reduction, of tumor growth after application of three cycles of LPEI-PEG-GE11/NIS followed by ^131^I. Mouse survival was prolonged in the therapy group, especially compared to the non-coding LPEI-PEG-GE11/antisenseNIS-treated control group, although without reaching statistical significance, despite the strong effects on tumor growth. Interestingly, while animals in the saline group had to be sacrificed due to compromised well-being owing to excessive tumor growth, the non-coding control group showed signs of ill health at much lower tumor volumes and had to be sacrificed. Similarly, effects on animal health were observed in the therapy group, though to a lower extent. We attribute this observation to toxicity of the LPEI-based conjugates, possibly combined with effects from ^131^I. Due to the stabilization of tumor growth in therapy animals, they fared better than the non-coding control group that was potentially affected by side effects from polyplex and radioiodide injection in addition to rapid tumor growth. To date, the use of LPEI-based polymers did not affect animal health in any of our previous studies, nor was viability of PDAC cell explants affected in the current study. LPEI has been shown to exhibit certain cytotoxic effects both *in vitro* and *in vivo* [37–41], though LPEI-based polyplexes have already been tested in a clinical trial for bladder cancer therapy and no adverse effects were reported [42]. Similarly, we have so far only encountered side effects from ^131^I in one previous study with the objective to radioablate mouse thyroids under intentional stimulation of thyroidal radioiodide uptake [43]. Symptoms developed with a delay of seven days after radioiodide application, while in the current study, animal health deteriorated from the beginning of treatment [43]. However, our earlier work was done in subcutaneous xenograft models, where tumor growth *per se* has no impact on animal health. In contrast, *Kras;p53* mice with their extremely aggressive pancreatic tumor growth and subsequent rapid health deterioration, seem to react more unfavorably to the polyplexes and/or radioiodide treatment. LPEI is seen as the “gold standard” for non-viral DNA delivery, as it shows such high transfection efficiency and flexibility at relatively low toxicity, compared to other viral and non-viral gene delivery approaches. To further refine our approach and solve the toxicity issue, we are currently developing sequence-defined polymers with higher biocompatibility for targeted *NIS* gene delivery [26].

In conclusion, our data clearly show the high potential of EGFR-targeted nanoparticle vectors to target the *NIS* gene to PDAC. After systemic application of LPEI-PEG-GE11/NIS, we were able to reach sufficient iodide concentrations at the tumor site to (1) produce a strong enough signal to image pancreatic tumors *in situ* and (2) provoke a therapeutic effect. Based on its role as potent and well characterized reporter gene, *NIS* allows non-invasive imaging and detailed characterization of *in vivo* biodistribution of functional NIS expression as an essential prerequisite for exact planning and monitoring of clinical gene therapy trials with the aim of individualization of the *NIS* gene therapy concept in the clinical setting.

## MATERIALS AND METHODS

### Establishment of genetically modified mice

Establishment of the *Kras;p53* (*Ptf1a^+/Cre^;Kras^+/LSL-G12D^;Trp53^loxP/loxP^*) and *Kras;p53;Egfr* (*Ptf1a^+/Cre^;Kras^+/LSL-G12D^;Trp53^loxP/loxP^;Egfr^fl/fl^*) strains has been described previously [4, 11, 12, 44, 45]. Mouse strains were maintained on a mixed C57BL/6;129/Sv background. Animals were kept under specific pathogen-free conditions with access to mouse chow and water *ad libitum*. Both male and female mice at 5-7.5 weeks of age were used for experiments. The experimental protocol was approved by the regional governmental commission for animals (Regierung von Oberbayern, Munich, Germany).

### Preparation and culture of PDAC cell explants

Cell explants from primary PDAC of *Kras;p53* and *Kras;p53;Egfr* mice were isolated as described previously [46] and cultured in DMEM high glucose medium (Invitrogen, Karlsruhe, Germany) supplemented with 10% fetal bovine serum (v/v; PAA, Colbe, Germany), 100 U/mL penicillin/100 μg/mL streptomycin (Invitrogen) and 1% non-essential amino acids (v/v; Invitrogen). Cells were maintained at 37°C and 5% CO_2_ in an incubator with 95% humidity. Cell culture medium was replaced every second day and explants were passaged at 85% confluency.

### Plasmid and polymer synthesis and polyplex formation

The human *NIS*-encoding plasmid and LPEI-based conjugates were cloned and synthesized, respectively, as described previously [17]. Plasmid DNA was condensed with polymers at indicated c/p ratios (w/w) in HEPES-buffered glucose (HBG: 20 mmol/L HEPES, 5% glucose (w/v), pH 7.4) as described previously [47] and incubated at room temperature for 20 min before use. Final DNA concentrations were 2 μg/mL for *in vitro* studies and 200 μg/mL for *in vivo* studies.

### Transient transfection

For *in vitro* transfection experiments, PDAC cell explants were grown to 60-80% confluency. Explants were incubated for 4 hours with polyplexes in the absence of serum and antibiotics followed by incubation with complete growth medium for 24 h. Either LPEI-PEG-GE11/NIS (EGFR-targeting of NIS due to the EGFR-specific ligand GE11), LPEI-PEG-Cys/NIS (no active targeting of NIS to EGFR, as the ligand GE11 is replaced by a cysteine residue), or LPEI-PEG-GE11 alone (polymer without NIS DNA) were added in c/p ratios as indicated. Transfection efficiency was evaluated by measurement of iodide uptake activity as described below. Transfections were done in triplicate for each separate explant.

### *In vitro* ^125^I uptake assay

Following transfections, iodide uptake of PDAC cell explants was determined at steady-state conditions as described previously [48, 49]. Results were normalized to cell viability that was measured using the commercially available MTS-assay (Promega, Mannheim, Germany) as described previously [50].

### Radioiodide uptake after systemic NIS gene transfer *in vivo*

For the proof-of-principle of NIS-mediated tumor-specific radioiodide accumulation *in vivo*, polyplexes (LPEI-PEG-GE11/NIS, c/p 0.8) were applied via the tail vein at a DNA dose of 2.5 mg/kg (50 μg DNA in 250 μL HBG). Mice received 18.5 MBq ^123^I (GE Healthcare, Braunschweig, Germany) intraperitoneally (i.p.) 24 h (n=9) or 48 h (n=7) after polyplex injection and radioiodide distribution was monitored by serial imaging on a gamma camera (Forte, ADAC Laboratories, Milpitas, CA, USA) equipped with a VXHR (ultra-high resolution) collimator as described previously [50]. Regions of interest were quantified and expressed as a fraction of the total amount of applied radionuclide per gram tumor tissue. The retention time within the tumor was determined by serial scanning after radioiodide injection. A subset of mice (n=2 for each time point) was pretreated i.p. with 2 mg of the competitive NIS inhibitor sodium perchlorate (NaClO_4_; Sigma-Aldrich, Taufkirchen, Germany) 30 min before ^123^I administration. Dosimetric calculations for ^131^I were made using the Medical Internal Radiation Dose (MIRD) technique and a RADAR dose factor (http://www.doseinfo-radar.com). In order to achieve better discrimination between uptake in the tumor and the adjacent stomach, 24 or 48 h after i.v. injection of polyplexes (LPEI-PEG-GE11/NIS, each time point n=5; LPEI-PEG-GE11/antisenseNIS, each time point n=1) mice received 10 MBq ^124^I (Perkin Elmer, Waltham, MA, USA) i.p. and radioiodide biodistribution was monitored by static acquisition 3 h post injection using a micro PET system (Inveon, Siemens Preclinical Solutions, Erlangen, Germany). Mean tumoral radioiodide uptake was calculated in MBq/mL by manually placing 3D regions of interest in the tumor.

### Analysis of NIS mRNA expression by quantitative real-time PCR

Total RNA was isolated from PDAC or non-target tissues (liver, lung) using the RNeasy Mini Kit (Qiagen, Hilden, Germany) according to the manufacturer's recommendations. Single-stranded oligo (dT)-primer cDNA was generated using Super Script III Reverse Transcriptase (Invitrogen). qPCR was performed with the cDNA from 1 μg RNA using SYBR Green PCR master mix (Qiagen) in a Rotor Gene 6000 (Corbett Research, Morthlake, New South Wales, Australia). The following primers were used: *NIS*, forward 5′-ACACCTTCTGGACCTTCGTG-3′, reverse 5′-GTCGCAGTCGGTGTAGAACA-3′ and *GAPDH*, forward 5′-GAGAAGGCTGGGGCTCATTT-3′, reverse 5′-CAGTGGGGACACGGAAGG-3′. Relative expression levels were calculated using the comparative ΔΔC_t_ method and internal *GAPDH* for normalization.

### Analysis of tissue sections

Immunohistochemical and immunofluorescence staining of NIS was performed using a mouse monoclonal antibody directed against human NIS (kindly provided by John C Morris, Mayo Clinic, Rochester, MN, USA) as described previously [20, 51].

### Radioiodide therapy

Starting when mice were around 30 d of age, tumor sizes were assessed weekly by high resolution MRI on a 3T clinical scanner (Philips Ingenia 3.0T; Royal Philips Electronics, Eindhoven, The Netherlands). Once tumors reached the inclusion size of 200-450 mm^3^, therapy trials were started. To this end, 48 h after systemic administration of LPEI-PEG-GE11/NIS or, as control, LPEI-PEG-GE11/antisenseNIS, a therapeutic dose of 55.5 MBq ^131^I (GE Healthcare) was administered i.p. (LPEI-PEG-GE11/NIS + ^131^I n=6; LPEI-PEG-GE11/antisenseNIS + ^131^I n=3). A second control group received saline only (n=4). The cycle consisting of systemic *NIS* gene transfer followed by radioiodide was repeated for a total of three times on days 0/2, 4/6 and 7/9. Mice from all groups were sacrificed when at least one endpoint criterion was reached. Endpoint criteria included a tumor volume >1000 mm^3^, a body weight loss >15%, as well as a number of general physical, clinical and behavioral criteria. Body condition was monitored by independent animal care personnel blind to treatment and hypothesis.

### Statistics

Results are reported as mean ± S.E.M., mean-fold change ± S.E.M. or, for survival plots, percent. Statistical significance was generally tested by two-tailed Student's t-test except for the therapy study. For tumor volumes, one-way ANOVA was performed, followed by Tukey's Honestly Significant Difference test. Statistical significance of Kaplan-Meier plots was analyzed by log-rank test. *p*-values <0.05 were considered statistically significant (*p<0.05; **p<0.01; ***p<0.001; n/s not significant).
